# Static Magnetic Field Reduces the Anticancer Effect of Hinokitiol on Melanoma Malignant Cells—Gene Expression and Redox Homeostasis Studies

**DOI:** 10.3390/ph17040430

**Published:** 2024-03-27

**Authors:** Agnieszka Synowiec-Wojtarowicz, Agata Krawczyk, Magdalena Kimsa-Dudek

**Affiliations:** Department of Nutrigenomics and Bromatology, Faculty of Pharmaceutical Sciences in Sosnowiec, Medical University of Silesia in Katowice, 8 Jednosci Street, 41-200 Sosnowiec, Poland; akrawczyk@sum.edu.pl (A.K.); mkimsa@sum.edu.pl (M.K.-D.)

**Keywords:** hinokitiol, static magnetic field, melanoma malignant, redox homeostasis, gene expression

## Abstract

Background: Melanoma malignant is characterized by a high mortality rate, accounting for as much as 65% of deaths caused by skin cancer. A potential strategy in cancer treatment may be the use of natural compounds, which include hinokitiol (β-Thujaplicin), a phenolic component of essential oils extracted from cypress trees. Many studies confirm that a high-induction SMF (static magnetic field) has anticancer effects and can be used as a non-invasive anticancer therapy in combination with or without drugs. Aim: The aim of this experiment was to evaluate the effect of a static magnetic field on melanoma cell cultures (C32 and COLO 829) treated with hinokitiol. Methods and Results: Melanoma cells were exposed to a static magnetic field of moderate induction and hinokitiol. The research included determining the activity of the antioxidant enzymes (SOD, GPx, and CAT) and MDA concentration as well as the gene expression profile. Conclusion: Hinokitiol disturbs the redox homeostasis of C32 and COLO 829 melanoma malignant cells. Moreover, a static magnetic field has a protective effect on melanoma malignant cells and abolishes the anticancer effect of hinokitiol.

## 1. Introduction

Malignant melanoma is characterized by a high mortality rate, which is due to the fact that this cancer is one of the most aggressive skin cancers with a high potential for metastasis. According to research, the high aggressiveness of melanoma results from genetic mutations and may be the result of increased expression of proteins causing rapid tumor growth and the formation of numerous infiltrates [1]. A potential strategy in cancer treatment may be the use of natural compounds, which include hinokitiol (β-Thujaplicin), a phenolic component of essential oils extracted from cypress trees. It was found that hinokitiol is a derivative of tropolone with high effectiveness, a broad spectrum of antiviral, antibacterial, antifungal, anti-inflammatory, and anticancer effects. Hinokitiol has been widely used in cosmetology as an ingredient of hair products, toothpastes, and bath cosmetics and as a food additive with antimicrobial properties. Hinokitiol has no toxic or carcinogenic effect in the body because it is not accumulated in our cells, tissues, or organs [2,3,4]. The literature reports indicate that hinokitiol has a broad spectrum of anticancer effects, including on melanoma malignant, liver, lung, and breast cancer cells. Its mechanism of action consists primarily in disturbing the redox balance of cells, inhibiting the cell cycle, and activating signaling pathways leading to programmed apoptosis of cancer cells [5,6]. In other studies, hinokitiol has found that its anticancer effects through several mechanisms, including MMP suppression, change in the activity of antioxidant enzymes (catalase and superoxide dismutase), caspases-9 and -3 activities, and induction of cytochrome c expression [7]. Research indicates that hinokitiol can protect against oxidative stress and restore transferrin function. TBHP-induced ferroptosis can be reversed with hinokitiol by inhibiting the JNK pathway. Moreover, studies have proven that hinokitiol can also induce cytotoxicity through its pro-oxidant effect: hinokitiol/transition metal complexes produce reactive oxygen species, which causes the inactivation of aconitase and the production of hydroxyl radicals, thus creating 8-hydroxy-2′-deoxyguanosine in DNA [8].

Also, due to the multidirectional positive effect of hinokitiol on the body and the fact that it is a zinc ionophore, a combination of zinc and hinokitiol was used with positive effect in the prevention and treatment of COVID-19 [9].

The common presence of a static magnetic field (SMF) in human life and its wide use in medicine necessitate the study of the biological impact of SMFs on cells and tissues and the determination of the mechanisms underlying the impact of SMFs on biological systems. Exposure to a static magnetic field can affect biological processes. Although SMFs have been found to be safe at the level of organs and cells, working people exposed to SMFs report many side effects [10]. Explaining the effect of SMFs on living cells is very difficult and ambiguous because the research results presented in the literature are different and depend on the type of cells examined, the source of the static magnetic field, and its induction. Scientific evidence suggests that SMFs can change cell orientation and modulate processes related to metabolism, proliferation, the structure of cell membranes, and modulate the level of gene expression in cells. Moreover, many studies suggest that disruption of the oxidation–reduction balance of cells leading to oxidative stress may result from increased exposure to an SMF [11,12,13]. Static magnetic fields have been extensively studied in cancer research as a potential adjunct to anticancer drug treatment. The results of these studies showed different effects of SMFs resulting mainly from the different induction of the applied field. Positive results of anticancer activity were confirmed, among others, for SMFs with induction exceeding 10 T [14].

Melanoma malignant is characterized by a high mortality rate, accounting for as much as 65% of deaths caused by skin cancer. Because malignant melanoma is often a difficult-to-treat cancer, we are looking for the possibility of joint action of physical factors, i.e., a static magnetic field and biologically active substances against melanoma cells. It turns out that such an effect depends on the chemical structure of the antioxidant substances used because some of them in combination with the field show a strong anticancer effect, while others lose their anticancer properties under the influence of SMFs. This may be due to the fact that the magnetic field can modify the structure of compounds. Such effects of an SMF may be unfavorable for patients due to the ubiquity of the magnetic field in everyday life, which in combination with treatment may result in its reduced effectiveness. Therefore, it is reasonable to continue searching for compounds that together with an SMF may have anticancer effects and to explain the mechanism of the negative impact of the field on substances with anticancer properties.

Difficulties in its treatment result from the very rapid development of the disease and the effective response of melanoma cells to external factors, which in many cases results in resistance to the therapy used. Therefore, the aim of the study was to assess the interaction of a static magnetic field on melanoma cell cultures (C32 and COLO 829) treated with hinokitiol. The influence of hinokitiol and SMFs on the gene expression encoding enzymes constituting the first line of antioxidant defense of the body was assessed, and it was checked whether changes at the molecular level correspond to changes at the protein level.

## 2. Results

### 2.1. Cytotoxicity of Hinokitiol

**Figure 1:**
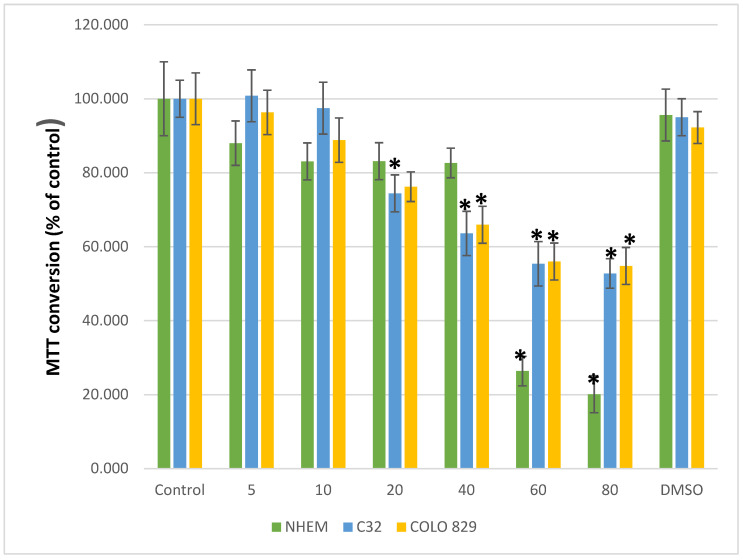
Cell viability by MTT test in melanoma malignant cells (COLO 829 and C32) and normal skin melanocytes (NHEM) that were treated with hinokitiol (between 5 and 80 μM for 24 h). Each bar represents the mean ± SD of two independent experiments. Statistical significance, * *p* < 0.05 vs. control.

The cytotoxicity of hinokitiol against melanoma malignant cells (C32 and COLO 829) and normal skin melanocytes (NHEM) was assessed in the concentration range of 5–80 µM. The concentration of 40 µM was selected for further research because it caused a statistically significant reduction in the number of viable cancer cells and was not cytotoxic to normal cells (Figure 1).

### 2.2. Effect of Hinokitiol and SMF on the Antioxidant Gene Expression

**Figure 2:**
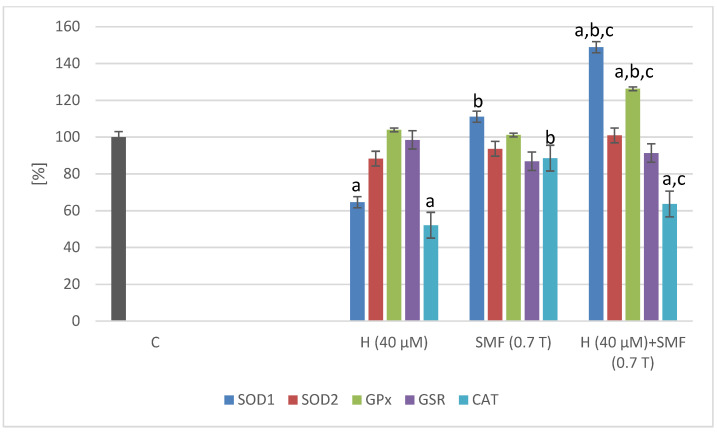
Effect of hinokitiol and SMF on the antioxidant gene expression in COLO 829 cell cultures. The results are presented as a percentage in relation to control (C) cultures; a *p* < 0.05 vs. C (control); b *p* < 0.05 vs. H (hinokitiol); and c *p* < 0.05 vs. SMF. C – control cultures without the addition of hinokitiol and not exposed to SMF and placed in 0T induction chambers.

**Figure 3:**
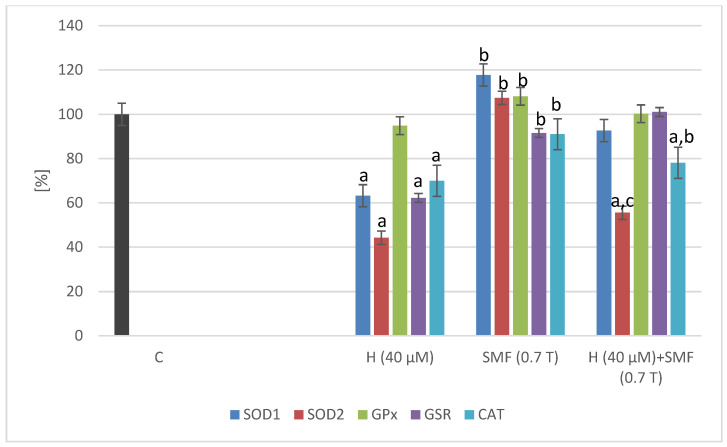
Effect of hinokitiol and SMF on the antioxidant gene expression in C32 cell cultures. The results are presented as a percentage in relation to control (C) cultures; a *p* < 0.05 vs. C (control); b *p* < 0.05 vs. H (hinokitiol); and c *p* < 0.05 vs. SMF. C – control cultures without the addition of hinokitiol and not exposed to SMF and placed in 0T induction chambers.

A static magnetic field with an induction of 0.7 T did not cause statistically significant changes in the expression of the tested genes (*SOD1*, *SOD2*, *GPx*, *CAT*, and *GSR*) in both C32 and COLO 829 cell cultures (Figure 2 and Figure 3). The addition of the hinokitiol to the COLO 829 cell cultures at a concentration of 40 µM resulted in a statistically significant decrease (*p* = 0.0012—*SOD1*; *p* = 0.02—*CAT*) in the expression of the *SOD1* and *CAT* genes compared to the control cell cultures. The addition of hinokitiol to C32 cell cultures resulted in a statistically significant reduction in the expression of *SOD1*, *SOD2*, *CAT,* and *GSR* (*p* = 0.007—*SOD1*; *p* = 0.033—*SOD2*; *p* = 0.008—*CAT*; *p* = 0.011—*GSR*) genes compared to control cultures. The co-exposure of the cells (C32 and COLO 829) to an SMF and hinokitiol normalized (COLO 829: *SOD1*, *GPx*, and *CAT*; C32: *SOD2* and *CAT*) the mRNA levels of the studied genes compared to the control cultures.

### 2.3. Effect of Hinokitiol and SMF on the Parameters of Redox Homeostasis

**Figure 4:**
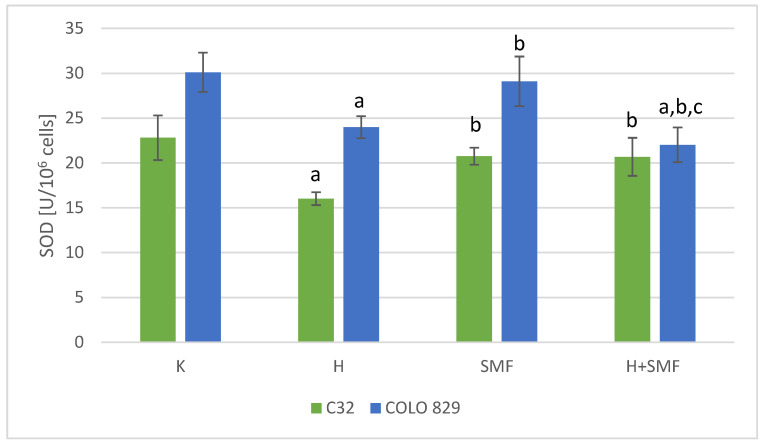
SOD activity in melanoma malignant cells co-exposed to hinokitiol and SMF. The results are presented as the mean ± SD; a *p* < 0.05 vs. K (control); b *p* < 0.05 vs. H (hinokitiol—40 µM); and c *p* < 0.05 vs. SMF (0.7 T).

**Figure 5:**
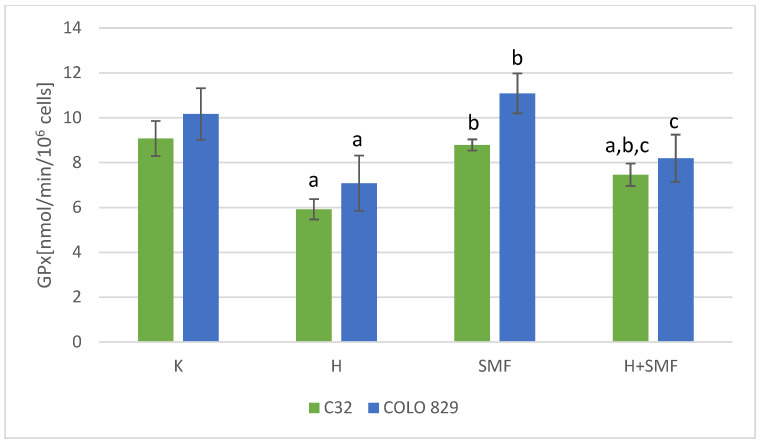
GPx activity in melanoma malignant cells co-exposed to hinokitiol and SMF. The results are presented as the mean ± SD; a *p* < 0.05 vs. K (control); b *p* < 0.05 vs. H (hinokitiol—40 µM); and c *p* < 0.05 vs. SMF (0.7 T).

**Figure 6:**
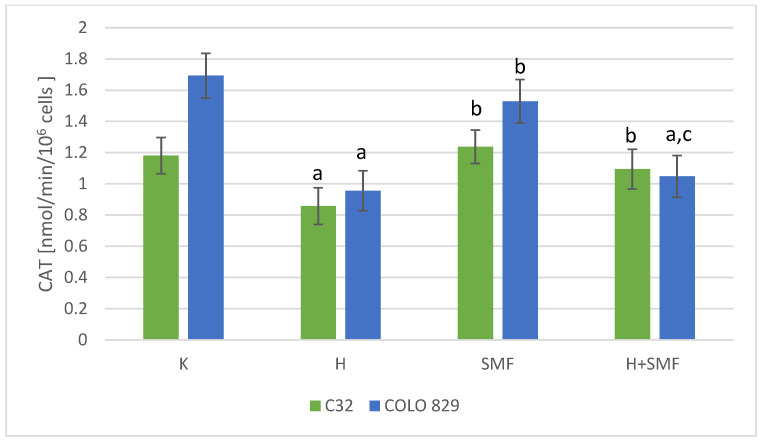
CAT activity in melanoma malignant cells co-exposed to hinokitiol and SMF. The results are presented as the mean ± SD; a *p* < 0.05 vs. K (control); b *p* < 0.05 vs. H (hinokitiol—40 µM); and c *p* < 0.05 vs. SMF (0.7 T).

**Figure 7:**
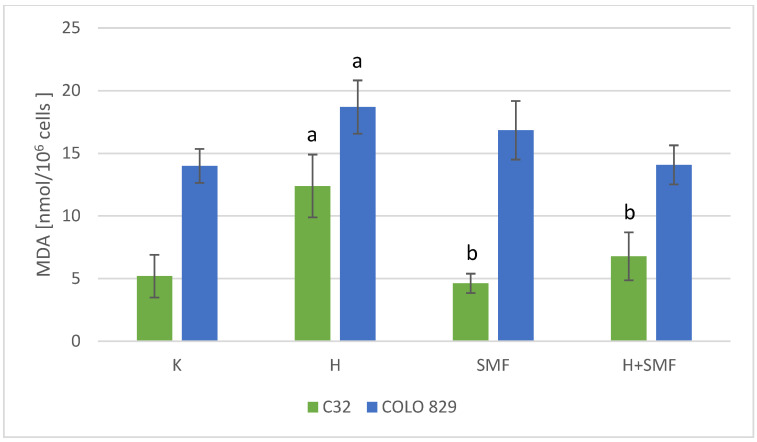
MDA concentration in melanoma malignant cells co-exposed to hinokitiol and SMF. The results are presented as the mean ± SD; a *p* < 0.05 vs. K (control); b *p* < 0.05 vs. H (hinokitiol—40 µM).

The activity of superoxide dismutase, glutathione peroxidase, and catalase, which are the main defense mechanisms of cells against the free radicals, in cultures of melanoma cells of both lines exposed to hinokitiol was statistically significantly lower in relation to cultures without the addition of hinokitiol (Figure 4, Figure 5 and Figure 6). The addition of hinokitiol to the culture of melanoma malignant cells resulted in a statistically significant decrease in superoxide dismutase activity by approximately 30% C32; 20% COLO 829 (*p* = 0.023 C32; *p* = 0.014 COLO 829); glutathione peroxidase by 35% C32 and 30% COLO 829 (*p* < 0.001 C32; *p* = 0.012 COLO 829); and catalase by 27% for C32 (*p* = 0.008) cell culture and 44% for COLO 829 (*p* < 0.001) cell culture compared to the control cell cultures. Exposure of both C32 and COLO 829 cells to a static magnetic field with an induction of 0.7 T did not cause statistically significant changes in the activity of the tested antioxidant defense enzymes (Figure 4, Figure 5, Figure 6 and Figure 7). However, simultaneous exposure of melanoma cells to hinokitiol at a concentration of 40 μM and a static magnetic field with an induction of 0.7 T resulted in the normalization of enzyme activity in relation to control cultures.

The study also determined the concentration of MDA, which is a marker of the lipid peroxidation process. In the cultures of both cell lines exposed to SMFs with an induction of 0.7 T, there were no statistically significant changes in the MDA concentration in relation to the control cultures (Figure 7). However, the addition of hinokitiol at a concentration of 40 μM to the culture of melanoma malignant cells resulted in a statistically significant increase in the MDA concentration by approximately 138% C32 and 34% COLO 829 (*p* < 0.001 C32; *p* = 0.002 COLO 829) in relation to control cultures. Moreover, simultaneous exposure of the tested cells to hinokitiol and SMF resulted in the normalization of MDA concentration and its reduction to values similar to those obtained for control cells.

## 3. Discussion

The ubiquity of a static magnetic field in human life may disturb the proper functioning of cells, tissues, and organs, which is why research on the influence of a static magnetic field is a very popular trend among scientists. The research results so far do not clearly determine whether an SMF has a positive or negative effect on our body. A static magnetic field has been used in the treatment of diseases of the musculoskeletal system, nervous system, and soft tissues for many years. The healing effect of SMFs results from its anti-inflammatory and anti-edematous properties and its participation in the process of soft tissue regeneration. Moreover, many studies confirm that high-induction SMFs have anticancer effects and can be used as a non-invasive anticancer therapy in combination with or without drugs. However, despite the positive effect of SMFs on the body, there are many studies confirming that magnetic fields may lead to excessive production of free radicals, which results in the induction of oxidative stress and disruption of the oxidation–reduction balance in the cell [15]. Our previous studies show that an SMF with an induction of 0.7 T does not cause disturbances in redox homeostasis in normal cells and cancer cells [16]. However, simultaneous exposure of cancer cells to an SMF and antioxidants commonly found in food and dietary supplements abolishes the anticancer effect of these substances [17,18]. Moreover, in a study by Wang et al. [14], it was found that a static magnetic field can inhibit the development of osteosarcoma, which is the effect of free radical processes dependent on iron ions. However, Yang et al. [19], examining lung cancer cells, observed a significant effect of a strong SMF (9.4 T) on the reduction in DNA synthesis and increased activity of the P-53 protein. The static magnetic field, based on the induction value, is classified as weak (<1 mT), moderate (1 mT–1 T), strong (1–5 T), and very strong (>5 T) [20]. According to the above division, the test chambers used in this study generated an SMF with moderate induction. The diversity of test results obtained by different researchers may result primarily from the source of the static magnetic field—special test chambers with permanent magnets were used in this study—as well as from the induction of the applied magnetic field.

Due to the low therapeutic potential of melanoma malignant cells, combined therapies containing drugs or substances with confirmed anticancer activity and physical factors such as a static magnetic field are being sought. The aim of the study was to assess the co-exposure of malignant melanoma cells to hinokitiol and SMF. Hinokitiol was selected for the study as a substance with confirmed anticancer activity, but so far there are no studies on whether therapeutic or everyday exposure to SMFs can modulate the anticancer properties of hinokitiol. In this experiment, hinokitiol at a concentration of 40 μM was used because it was found to be cytotoxic to melanoma cells (C32, COLO 829) but not toxic to normal skin melanocytes (NHEM). Hang et al. [4], examining the level of metalloproteinases in melanoma cells (B16-F10), found that the anticancer mechanism of hinokitiol may be related to blocking the signaling pathways and transcription factors NF-κB and c-Jun. This action contributes to reducing the number of metastases and inhibiting the growth of the examined cancer cells. Shih et al. [21] showed that hinokitiol at a concentration of 200 μM has anticancer activity against oral squamous cell carcinoma cells, but has no cytotoxic effect on normal oral keratinocytes.

The research results so far indicate that compounds with antioxidant activity used in high concentrations or doses may have a pro-oxidant effect. The anticancer effect of many bioactive substances is related to their pro-oxidant effect on cancer cells. Moreover, it was found that when the amount of reactive oxygen species in the cell is low, the defense mechanisms are activated, the expression of genes encoding first-line defense enzymes increases, and the activity of enzymes such as SOD, GPx, and CAT increases. However, a very large amount of free radicals leads to impairment of defense mechanisms, resulting in reduced gene expression and enzyme activity. Complete failure of defense mechanisms leads to cell death [22,23]. In this study, we found that hinokitiol at a concentration of 40 μM reduced the activity of antioxidant enzymes: superoxide dismutase, glutathione peroxidase, and catalase in both C32 and COLO 829 cell cultures. Changes at the protein level correspond to changes at the molecular level, where a decrease in the expression of *SOD1, SOD2, GPx*, and *CAT* genes was found. The anticancer properties of hinokitiol have been confirmed, among others, in relation to prostate cancer, where Liu and Yamauchi [24] found disruption of androgen receptor signaling. In turn, in the study by Wang et al. [25], it was found that hinokitiol induces autophagy in breast cancer cells. While Jayakumar et al. [8] were examining adenocarcinoma cells, they found that hinokitiol inhibits metalloproteinases and induces cell apoptosis and antioxidant enzymes in the tested cells. The chemical structure of hinokitiol, and more specifically the presence of the α-hydroxyketone structure, demonstrates a strong ability to chelate iron ions. In turn, the increase in intracellular iron in cancer cells plays a key role in the proliferation of these cells. Iron deprivation strategies, such as iron chelators or reducing the level of iron regulatory protein-2 (IRP2), may inhibit the growth of cancer cells [26,27]. In a study conducted by Quin et al. [2], it was found that hinokitiol, by activating the lipid peroxidation process in cancer cells leading to DNA and cell damage, may be effective in supporting the treatment of cancer. In our studies, we also determined the concentration of MDA and found that hinokitiol causes an increase in MDA concentration in relation to control cultures in both C32 and COLO 829 cells.

The main aim of our research was to check whether a static magnetic field with an induction of 0.7 T can modify the anticancer effect of hinokitiol on melanoma malignant cells, both at the protein and molecular levels. We found that the SMF has a protective effect on cancer cells and normalizes the activity of antioxidant enzymes, MDA concentration, and the expression of the tested genes to values similar to those of control cultures. We obtained similar results in our previous studies, in which an SMF abolished the anticancer properties of resveratrol [28]. In contrast, the study by Yang et al. [19] found that the 88 h effects of an SMF treatment with 9.4 T induction depend on the direction of SMF action. When the SMF direction is vertically upward, it causes significant inhibition of lung cancer cell growth (A549). Moreover, researchers suggest that the direction of the SMF may cause differences in biological effects on cells. Many studies confirm that an upward SMF 0.2—1 T may have cytotoxic effects on cancer cells, while the same downward SMF does not have such an effect [29]. Also, in the study by Wang et al. [14], it was found that an SMF with 12 T induction causes an intracellular increase in iron concentration in osteosarcoma cells, which leads to an increase in the amount of reactive oxygen species and, as a result, inhibits the proliferation of osteosarcoma cells. Moreover, they found that a 12 T SMF may enhance the anticancer effects of drugs such as cisplatin and sorafenib. Despite many studies confirming the anticancer effect of SMFs, especially those with high induction, there are also studies confirming that SMFs with weak to moderate induction can reduce the level of ROS in many cell lines. Reactive oxygen and nitrogen species play an important role in the body’s natural defense against cancer, mainly through intracellular signaling pathways, but the overproduction of free radicals may damage the ion channels of cancer cells, leading to their apoptosis [30]. Li et al. [31], treating human hepatoma cells (BEL-7402 and HepG2) with an SMF with an induction of 0.2 T, found significant apoptosis of BEL-7402 cells, but a similar effect was not observed in HepG2 cells. The use of an SMF resulted in a decrease in the expression of Bcl-2 and caspase-8 and an increase in the expression of caspases-3 and -9. As you can see, the research results so far are inconsistent, and researchers have hypothesized that the biological effects of an SMF depend on whether exposure to the field occurs alone or in combination with chemical factors because the SMF itself is considered harmless to cells and tissues, with the exception of fields with high induction. Moreover, the observed differences in study results can be explained by differences in the parameters of exposure to the SMF (induction, field source, and exposure duration), differences in the sensitivity of the cell types tested, and interactions between the SMF and the chemical agents used [32].

## 4. Materials and Methods

### 4.1. Chemicals

Sigma Aldrich (St. Louis, MO, USA): antibiotics, (penicillin and amphotericin B), DMSO (dimethyl sulfoxide), hinokitiol, lysis buffer, (protease inhibitor and phosphatase inhibitor), cytotoxicity test, and MTT (3-(4,5-dimethylthiazol-2-yl)-2,5 diphenyltetrazolium bromide).

Lonza (Basel, Switzerland): cell medium: DMEM (Dulbecco’s Modified Eagle Medium), fetal bovine serum, trypsin/EDTA solution, and PBS (buffered saline solution).

Invitrogen (Waltham, MA, USA): 0.4% trypan blue and TRIzol reagent.

### 4.2. Melanoma Cell Culture

The C32 and COLO 829 melanoma malignant cells used in the study were purchased from ATCC (CRL-1585, CRL-1974; Manassas, VA, USA). DMEM culture medium enriched with 10% fetal bovine serum and antibiotics, amphotericin B (0.25 mg/mL) and penicillin (10,000 U/mL), was used for cell culture. The cells were grown in culture flasks, which were placed in an incubator at 37 °C in 5% CO_2_. After completion of culture, melanoma cells were stained with 0.4% trypan blue, and their viability was assessed using the Countess TM Automated Cell Counter (Invitrogen, Carlsbad, CA, USA). In the experiment, melanoma cells around the 5th passage were used and only those that were characterized by high viability.

### 4.3. Hinokitiol Cytotoxicity

For the cytotoxicity of hinokitiol at a concentration of 5 to 80 µM on C32 and COLO 829 cells, MTT—3-[4,5-dimethylthiazol-2-yl]-2,5-diphenyltetrazolium bromide method—was used [33]. The effect of hinokitiol (5–80 µM) on the culture of normal skin melanocytes (NHEM) was also examined. Hinokitiol was weighed and dissolved in DMSO, the concentration of which was not higher than 1%. A stock solution was then prepared in the appropriate C32, COLO 829, or NHEM cell culture medium. In the MTT assay, the ability of cells to convert MTT indicated mitochondrial activity and, consequently, cell viability. C32, COLO 829, and NHEM cells were grown in 96-well culture plates at a density of 5000 cells/well. After 24 h of hinokitiol treatment, the medium was filtered, and MTT (1 mg/mL) was added and then incubated for 3 h (at 37 °C). Then, 100 μL of DMSO was added to each well, and the absorbance was read at 540 nm on a Wallace 1420 VICTOR microplate reader (PerkinElmer, Waltham, MA, USA).

### 4.4. Static Magnetic Field Exposure

The tested C32 and COLO 829 cells were treated with hinokitiol at a concentration of 40 µM and exposed to a static magnetic field with an induction of 0.7 T. The concentration of hinokitiol was selected based on cytotoxicity assessment. A concentration of 40 µM was selected for the experiment because it was cytotoxic to melanoma malignant cells (C32 and COLO 829) but was not cytotoxic to normal skin melanocytes (NHEM).

The construction of the magnetic chamber emitting a static magnetic field, in which melanoma malignant cells were cultured, was described in detail in previous studies [34,35]. Melanoma cells were cultured for 24 h in culture flasks, which were placed in test chambers of a static magnetic field with an induction of 0.7 T in an incubator, under conditions of 37 °C and 5% CO_2_. Then, the cultures were liquidated, and the cells were counted in a cell counter after staining the cells with 0.4% trypan blue.

### 4.5. Preparing the Cell Lysates

After 24 h of the experiment, the tested cancer cells were lysed in lysis buffer. First, the cells were treated with trypsin/EDTA solution, neutralized with culture medium, centrifuged, and the obtained pellets were used to prepare lysates. Lysis buffer was prepared in 10 mL of PBS solution by weighing 14 mg of protease inhibitor and adding 100 μL of phosphatase inhibitor. Cell pellets obtained after centrifugation were then dissolved in lysis buffer so that 100 µL of buffer was added for each million living cells. The lysates obtained in this way were incubated for 30 min in liquid nitrogen and then stored at −80 °C until the tests were performed.

### 4.6. Biochemical Assays

Before starting the biochemical determinations, the stored melanoma cell lysates were thawed, centrifuged, and then the supernatant was collected, in which SOD, GPx, CAT activity, and MDA concentration were determined. All obtained results were converted to 10^6^ cells.

#### 4.6.1. Determination of the Activity of Antioxidant Enzymes

The activity of SOD, GPx, and CAT antioxidant enzymes was determined using test kits from Cayman Chemical (Cayman Chemical, Ann Arbor, MI, USA) and performed according to the protocol provided by the manufacturer. A detailed description of the tests performed was presented in previous works [28].

#### 4.6.2. Lipid Peroxidation Assay

The concentration of a marker of the lipid peroxidation process: MDA was also tested using the Bioxytech MDA-586 test kit (OxisResearch, Portland, OR, USA). The assay was performed according to the protocol provided by the manufacturer, and the absorbance of the reaction product was measured at 586 nm.

### 4.7. RNA Extraction

Total RNA was extracted using a TRIzol reagent [36]. The quality of the RNA extract was evaluated using agarose gel electrophoresis, but total RNA concentration was determined using MaestroNano MN-913 spectrophotometer (MaestroGen Inc., Las Vegas, NV, USA). COLO 829 cells were additionally purified of melanin, which can be performed by a PCR inhibitor, using OneStep™ PCR Inhibitor Removal Kit from Zymo Research (Irvine, CA, USA). Purification was performed according to the kit manufacturer’s protocol.

#### Quantitative Real-Time RT-qPCR Assay

The gene expressions of SOD1 (superoxide dismutase 1), SOD2 (superoxide dismutase 2), GSR (glutathione reductase), CAT (catalase), GPx1 (glutathione peroxidase 1), and ACTB (β-actin) were evaluated using the real-time RT-qPCR method (GoTaq^®^ 1-Step RT-qPCR System kit; Promega, Madison, WI, USA), specific primers (Table 1), and a LightCycler^®^ 480 Instrument II (Roche Life Science, Basel, Switzerland). The reaction parameters were as follows: reverse transcription—37 °C, 15 min; activation—95 °C, 10 min, 40 cycles; denaturation—95 °C, 10 s; annealing and data collection—60 °C, 30 s; and extension—72 °C, 30 s.

Samples were analyzed three times. The endogenous positive control was β-actin, but the negative controls were wells without a template [37].

### 4.8. Statistical Analyses

The results of all measurements were presented as the mean + standard deviation. ANOVA analysis and Tukey’s post hoc test were performed to evaluate the results of the experiment using STATISTICA 13.0, and the statistical significance was defined at *p* < 0.05.

## 5. Conclusions

These studies have shown that hinokitiol has anticancer effects by disturbing the oxidation–reduction homeostasis of melanoma cells. However, a static magnetic field abolishes the anticancer effect of hinokitiol and protects melanoma cells. This may be due to the fact that the magnetic field can modify the structure of compounds. Such effects of an SMF may be unfavorable for patients due to the ubiquity of the magnetic field in everyday life, which in combination with treatment may result in its reduced effectiveness. Therefore, it is reasonable to explain the mechanism of the negative impact of the field on substances with anticancer properties.

## Figures and Tables

**Table 1:** Characteristic of primers used for real-time RT-qPCR.

Gene	Sequence of Primers	Amplicon Length (bp)
*SOD1*	Forward: 5′-TTGGGCAATGTGACTGCTGACAAA-3′ Reverse: 5′-GGGCGATCCCAATTACACCACAA-3′	208
*SOD2*	Forward: 5′-CTGATTTGGACAAGCAGCAA-3′ Reverse: 5′-CTGGACAAACCTCAGCCCTA-3	199
*GSR*	Forward: 5′-AGAAATCATCCGTGGCCATGCA-3′ Reverse: 5′-ACCAACAATGACGCTGCGGC-3	214
*CAT*	Forward: 5′-CCTATCCTGACACTCACCGCCATCG-3′ Reverse: 5′-GGATGCTGTGCTCCAGGGCAGA-3	201
*GPx1*	Forward: 5′-AATGTGGCGTCCCTCTGAGGCA-3′ Reverse: 5′-GCTCGTTCATCTGGGTGTAGTCCCG-3′	55
*ACTB*	Forward: 5′-TCACCCACACTGTGCCCATCTACGA-3′ Reverse: 5′-CAGCGGAACCGCTCATTGCCAATGG-3′	295

## Data Availability

Data is contained within the article.
